# *Oxo*-aglaiastatin-Mediated Inhibition of Translation Initiation

**DOI:** 10.1038/s41598-018-37666-5

**Published:** 2019-02-04

**Authors:** Rayelle Itoua Maïga, Regina Cencic, Jennifer Chu, Daniel D. Waller, Lauren E. Brown, William G. Devine, Wenhan Zhang, Michael Sebag, John A. Porco Jr., Jerry Pelletier

**Affiliations:** 1 Department of Biochemistry, McGill University, Montreal, Québec H3G 1Y6 Canada; 2 Department of Chemistry and Center for Molecular Discovery (BU-CMD), Boston University, Boston, MA 02215 USA; 3 Department of Medicine, McGill University, Montreal, Québec H3G 1Y6 Canada; 4 Department of Oncology, McGill University, Montreal, Québec H3G 1Y6 Canada; 5 Rosalind & Morris Goodman Cancer Research Centre, McGill University, Montreal, Québec H3A 1A3 Canada

## Abstract

Translation is a highly regulated process that is perturbed in human cancers, often through activation of the PI3K/mTOR pathway which impacts directly on the ribosome recruitment phase of translation initiation. While significant research has focused on “drugging” components of the PI3K/mTOR network, efforts have also been directed towards inhibiting eukaryotic initiation factor (eIF) 4F-dependent translation. Small molecule inhibitors of this complex have been identified, characterized, and used to validate the rationale of targeting this step to curtail tumor cell growth and modulate chemotherapy response. One such class of compounds are the rocaglates, secondary metabolites from the plant genus *Aglaia*, which target the RNA helicase subunit of eIF4F, eIF4A. Here we explore the ability of synthetic derivatives of aglaiastatins and an aglaroxin derivative to target the translation process *in vitro* and *in vivo* and find the synthetic derivative *oxo*-aglaiastatin to possess such activity. *Oxo*-aglaiastatin inhibited translation *in vitro* and *in vivo* and synergized with doxorubicin, ABT-199 (a Bcl-2 antagonist), and dexamethasone when tested on hematological cancer cells. The biological activity of *oxo*-aglaiastatin was shown to be a consequence of inhibiting eIF4A1 activity.

## Introduction

Translation initiation in eukaryotes is a highly regulated process. A critical control point involves eukaryotic initiation factor (eIF) 4F, a complex that recruits the 40 S ribosome (and associated factors) to mRNA 5′ ends^1,2^. eIF4F is a heterotrimeric complex that binds the mRNA cap structure *via* its eIF4E subunit and utilizes its eIF4A helicase subunit to unwind local mRNA secondary structure in preparation for ribosome binding. eIF4F assembly falls under the governance of the PI3K/mTOR pathway, a signalling cascade usurped in the majority of human cancers - making it an attractive target for therapeutic development. It has been shown that eIF4E can exist in two distinct complexes, one as a component of eIF4F and the second, in complex with one of three repressor proteins known as eIF4E-binding proteins (4E-BP). Activation of mTOR leads to phosphorylation of 4E-BP, disrupting its association with eIF4E and increasing levels of eIF4F^1,2^. Alterations in eIF4F levels are associated with a selective change in the translation of choice mRNAs, several of which encode for activities that fuel the Hallmarks of Cancer^3^. Strategies that aim to dampen eIF4F levels or activity are currently being explored as anti-neoplastic agents and show promising activity in pre-clinical models^3^.

Among the small molecules found to inhibit eIF4F activity, rocaglates have shown impressive potency and exert their effects through the selective inhibition of eIF4A^4,5^. They increase the binding of eIF4A to polypurine-enriched RNA sequences and cause depletion of eIF4A from the eIF4F complex^6–8^. Several rocaglates have been shown to exhibit anti-cancer activity *ex vivo* and *in vivo* in several pre-clinical mouse cancer models^6,9–11^. At doses that partially inhibit translation, they exert selective changes to the translatome^8,12,13^.

**Figure 1:**
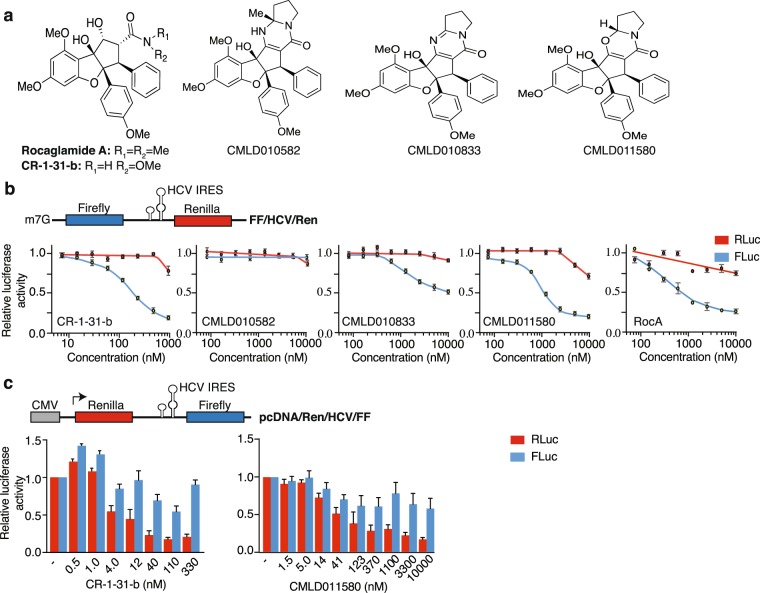
Aglaiastatin-mediated inhibition of cap-dependent translation. (**a**) Chemical structure of Roc A, CR-1-31-b, aglaiastatins (CMLD010582, CMLD011580) and aglaroxin (CMLD010833) used in this study. (**b**) *Top:* Schematic representation of the FF/HCV/Ren reporter mRNA used herein. *Bottom:* Assessment of cap- and HCV-mediated translation in the presence of the indicated compound concentrations in Krebs-2 extracts as indicated in the Materials and Methods. Luciferase activity results are expressed relative to values obtained in the presence of DMSO controls. Results are expressed as mean ± SEM of 4 biological replicates. (**c**) Assessment of CMLD011580 activity in HEK293 cells. *Top:* Schematic representation of the pcDNA/Ren/HCV/FF expression vector. *Bottom:* Effect of CMLD011580 on cap-dependent and HCV IRES–mediated translation in HEK293 cells transfected with pcDNA/Ren/HCV/FF. Luciferase activity is expressed relative to values obtained in DMSO-treated cells and is the mean ± SEM of 3 biological replicates.

Rocaglates are exclusive products of plants from the *Aglaia* (Meliaceae) genus. These plants produce several cyclopenta[*b*]benzofuran-containing metabolites, among which rocaglamide (Roc) A (Fig. 1a) was the first isolated active compound and shown to exhibit anti-leukemic activity in mice^14^. Since then, a large number of naturally occurring and synthetic rocaglamide derivatives (>100) have been characterized^15–17^. These fall within one of three classes based on their core structures: (i) cyclopenta[*bc*]benzopyrans (aka thapsakins), (ii) benzo[*b*]oxepines (aka thapoxepines) and, (iii) cyclopenta[*b*]benzofurans (aka rocaglates or flavaglines) (Suppl. Fig. 1). Among these metabolites, it is only the latter that have activity towards eIF4A and inhibit translation initiation^7^. Additionally, synthetic *aza*-rocaglates, in which the benzofuran ring oxygen is replaced by a nitrogen, have been synthesized and do not inhibit translation - highlighting the critical nature of the furan core for activity (Suppl. Fig. 1)^18^. One of the most studied rocaglates, silvestrol, exhibits potent anti-proliferative activity against many cancer cell lines *in vitro* and *in vivo* in xenograft models (reviewed in ref.^3^). Structure-activity relationship studies, facilitated by the development of an enantioselective synthesis approach^19^ have led to the identification of a synthetic derivative, (−)-CR-1-31-b (Fig. 1a) - a hydroxamate-containing rocaglamide with improved biological activity and anti-cancer properties^20^. Among the cyclopenta[*b*]benzofuran metabolites, aglaiastatins and aglaroxins have not been as extensively characterized (Suppl. Fig. 1). These molecules differ from rocaglates by the presence of a pyrimidone unit fused to the cyclopenta[*b*]benzofuran core while retaining the carbonyl and tertiary amine functional groups that could participate in target recognition. Herein, we document the inhibitory effect of select synthetic aglaiastatin derivatives towards translation and compare their relative activity to previously studied rocaglates.

## Results

### Assessment of *In Vitro* Activity

We undertook a comparative assessment of the synthetic, racemic aglaiastatin derivative (CMLD010582), the synthetic derivative (+)-*oxo*-aglaiastatin (CMLD011580), and (−)-aglaroxin C (CMLD010833) to the two active rocaglates, (−)-CR-1-31-b and (−)-RocA (Fig. 1a). CMLD010582 was synthesized as a potential stable analogue of aglaiastatin, a compound known to undergo facile oxidation to the pyrimidinone aglaroxin C^21^. Our initial comparison involved scoring for translation inhibitory activity *in vitro* in Krebs-2 extracts programmed with a FF/HCV/Ren bicistronic mRNA (Fig. 1b). This reporter encodes for firefly luciferase (FLuc) which reports on cap-dependent protein synthesis and renilla luciferase (RLuc) which is driven by the hepatitis C viral (HCV) internal ribosome entry site (IRES) and recruits ribosomes in an eIF4F-independent manner. Among the tested compounds, (−)-CR-1-31-b was the most potent showing an IC_50_ of ~100–200 nM towards inhibition of cap-dependent firefly production, while affecting renilla expression only at the highest tested concentration (Fig. 1b). CMLD010582 was ineffective at inhibiting cap- or HCV IRES-driven translation. CMLD010833 displayed an IC_50_ of ~10 μM towards firefly production, while not affecting renilla synthesis. CMLD011580 blocked firefly production with an IC_50_ of ~1 μM, a ~5–10-fold lower potency compared to (−)-CR-1-31-b but only ~1.5-fold lower than RocA (IC_50_ of ~700 nM) (Fig. 1b).

CMLD011580 also inhibited cap-dependent translation in rabbit reticulocyte lysates and wheat germ extracts (Suppl. Fig. 2a,b). When tested in HEK293 cells transfected with a Ren/HCV/FF expression vector, CMLD011580 exhibited an IC_50_ = ~41 nM, compared to (−)-CR-1-31-b which showed an IC_50_ = ~4 nM towards inhibition of cap-dependent renilla luciferase production (Fig. 1c). Similar to (−)-CR-1-31-b, acute exposure of cells to CMLD011580 blocked global ^35^S-methionine incorporation and showed no impact on RNA transcription (Suppl. Fig. 2c). As shown for other rocaglates^22,23^, CR-1-31-b and CMLD011580 induced cell cycle arrest at the G2/M boundary (Suppl. Fig. 2d). Also, as previously shown for silvestrol^5^, CR-1-31-b and CMLD011580 slightly stimulated eIF4A RNA helicase activity (Suppl. Fig. 2e) (see Discussion). Collectively, these results indicate that the (+)-*oxo*-aglaiastatin CMLD011580 selectively inhibits cap-dependent translation *in vitro* and in cells.

### *Oxo*-aglaiastatin targets eIF4A1

**Figure 2:**
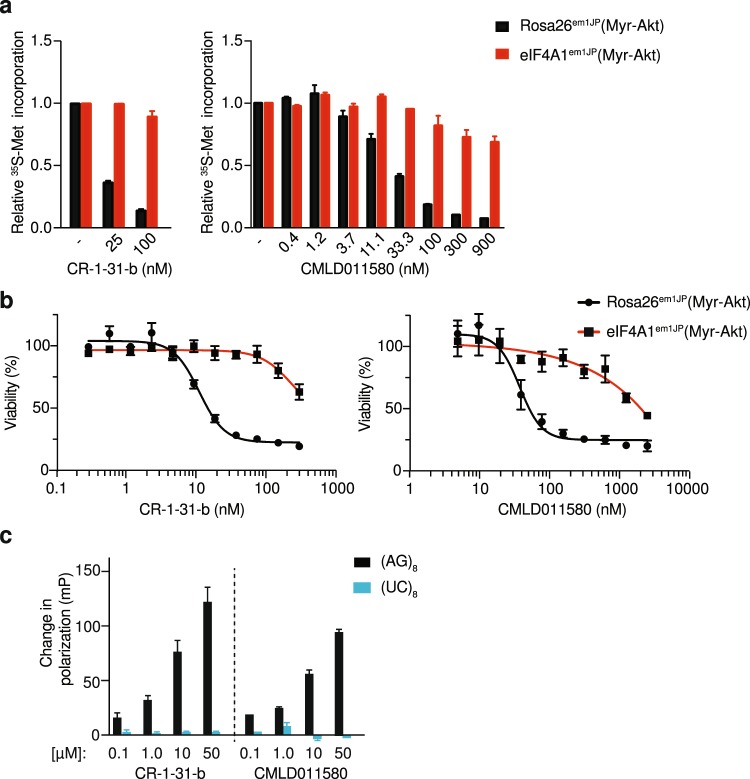
CMLD011580 targets eIF4A1. (a) Dose-dependent inhibition of protein synthesis *in vivo* by CMLD011580 is eIF4A1-dependent. Cells were incubated with the indicated concentrations of compound for 1 hour, and [^35^S]-methionine was added 15 minutes before the end of the incubation. The rate of [^35^S]-methionine incorporation is expressed relative to that of cells treated with vehicle (DMSO). Results are expressed as the mean ± SEM of 3 biological replicates. (**b**) Cellular toxicity of CMLD011580 is eIF4A1-dependent. Cells were incubated in the presence of the indicated concentrations of CMLD011580 or (−)-CR-1-31-b for 48 hrs, after which the percentage of viable cells was measured. Results are expressed as the mean ± SEM of 3 biological replicates. (**c**) Stimulation of eIF4A1:RNA binding by (−)-CR-1-31-b or CMLD011580. Fluorescently labelled RNA (16-mers) containing (AG)_8_ or (UC)_8_ repeats were used as probes in these experiments. Shown is the change in fluorescent polarization obtained in the presence of indicated compound concentration, above what was observed in the presence of DMSO. Results are expressed as the mean ± SEM of 3 biological replicates.

To determine if the observed inhibition of translation by CMLD011580 was a consequence of perturbing eIF4A activity, we took advantage of CRISPR-modified cells harboring engineered rocaglate-resistant eIF4A1 (F163L) alleles^5^. As previously reported, translation in eIF4A1^em1JP^ (Myr-Akt) cells harboring the eIF4A1 (F163L) alleles is resistant to inhibition by CR-1-31-b, whereas in the control Rosa26^em1JP^ (Myr-Akt) cells, protein synthesis is sensitive to (−)-CR-1-31-b (Fig. 2a, left panel). A similar response was observed when these two cell lines were incubated in the presence of increasing concentrations of CMLD011580 (Fig. 2a, right panel). Cells expressing the eIF4A1^F163L^ mutant protein were resistant to protein synthesis inhibition by CMLD011580 at concentrations up to 900 nM. The toxicity of (−)-CR-1-31-b and CMLD011580 was also substantially dampened in eIF4A1^em1JP^ (Myr-Akt) cells compared to Rosa26^em1JP^ (Myr-Akt) cells (Fig. 2b). CMLD011580 exhibited an IC_50_ for cytotoxicity of ~2 μM and 50 nM towards eIF4A1^em1JP^ (Myr-Akt) and Rosa26^em1JP^ (Myr-Akt) cells, respectively. For (−)-CR-1-31-b, these were in the range of >300 nM and ~15 nM respectively. RocA has been shown to stimulate binding of eIF4A1 to RNAs harboring polypurine sequences^8^. (−)-CR-1-31-b and CMLD011580 also demonstrated such a sequence-dependent stimulation with eIF4A1 in presence of a (AG)_8_-repeat RNA. This effect was not observed when a (UC)_8_-repeat RNA was used as the target ligand, indicating that CMLD011580 -mediated clamping of eIF4A1 is polypurine-selective (Fig. 2c).

### Assessment of CMLD011580 activity *in vivo*

**Figure 3:**
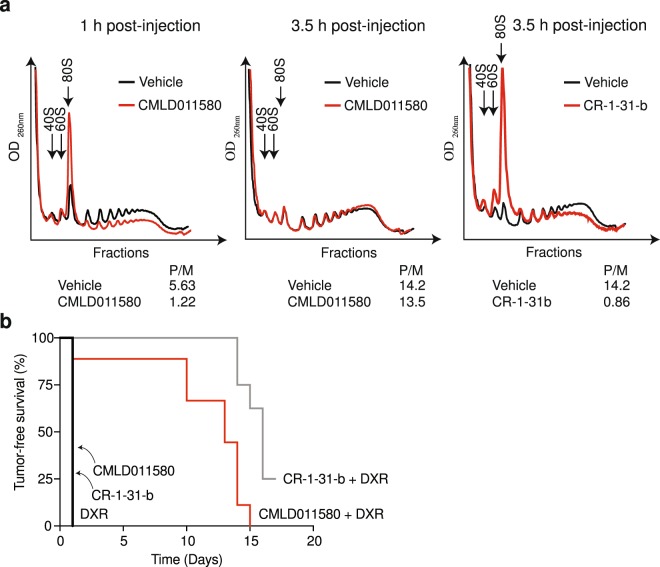
*In vivo* activity of CMLD011580. (**a**) Assessment of liver polysomes following administration of CMLD011580 in mice. Liver polysomes were isolated following intraperitoneal injection of mice with vehicle or 0.2 mg/kg CMLD011580 or (−)-CR-1-31-b at the indicated time points. (**b**) Kaplan-Meier plot illustrating the tumor-free survival of *Myr-Akt/Eμ-Myc* lymphoma bearing mice to the indicated therapeutic regimen. Tumor-free survival was assessed by measuring the time between complete tumor regression following drug administration and the reappearance of tumors in the inguinal lymph nodes. Each cohort had 5–10 mice. p < 0.001 for CMLD011580 + DXR versus DXR.

Preliminary pharmacological assessment of CMLD011580 indicated that it had a plasma half-life of ~60 min, was significantly bound to plasma (96% bound after 4 hours), and showed a high clearance rate by mouse liver microsomes (Table 1). We therefore assessed if CMLD011580 could block translation *in vivo* by monitoring the ability of this compound to disrupt polysomes in mouse livers. Polysomes were analyzed 1 and 3.5 h following intraperitoneal administration of CMLD011580 and revealed suppression of translation at 1 h post-injection, as visualized by the redistribution of a significant proportion of polysomes into free ribosomes (Fig. 3a). This effect was significantly reversed by 3 h post-injection, although it was sustained with  CR-1-31-b (Fig. 3a), which has a longer serum half-life (100% of compound remaining after 3 hrs^20^). These results indicate that CMLD011580 can inhibit protein synthesis *in vivo*, but the effects are not as long lasting as with CR-1-31-b. We saw no signs of toxicity associated with sustained injections of CMLD011580, as assessed by body weight measurements and grooming behaviour of the animals (Suppl. Fig. 3a). Additionally, we found that CMLD011580 is not a substrate for the multi-drug transporter, Pgp-1, displaying the same IC_50_ against HeLa cells lacking or over-expressing Pgp-1, in contrast to silvestrol, where the Pgp-1 expressing HeLa cells were ~100-fold more resistant (Suppl. Fig. 3b,c).

**Table 1:** *In vitro* assessment of CMLD011580 pharmacokinetic properties.

	CMLD011580
Plasma stability T_1/2_ (min)	63.4
Mean Plasma Fraction bound (%)	96.8
Microsomal clearance T_1/2_ (min)	5.09

All pharmacokinetic studies were performed by Cyprotex (United States) following standard protocols.

### CMLD011580 synergizes with doxorubicin

We then took advantage of the Eμ-Myc lymphoma model to assess the *in vivo* chemosensitization potential of CMLD011580. Eμ-Myc mice harbor a transgene in which c-MYC expression is driven by the IgH enhancer and invariably develop B cell lymphomas^24^. Specific genetic lesions can be introduced into this model with the aim of defining tumor initiation events and response to chemotherapy^25^. Mice bearing *Myr-Akt/Eμ-Myc* tumors, which exhibit deregulated mTORC1 activity and an increased dependency on eIF4F^26^, received either CMLD011580, CR-1-31-b, doxorubicin (DXR), or a combination of DXR with either compound (Fig. 3b). None of the tested compounds lead to tumor regression when delivered as single agents, however both CMLD011580 and (−)-CR-1-31-b synergized with DXR to significantly extend tumor-free survival. These results indicate that despite the shortened serum life of CMLD011580 compared to CR-1-31-b, the former compound is effective at synergizing with DXR, indicating that a transient inhibition of protein synthesis *in vivo* is sufficient for this effect.

### CMLD011580 synergizes with the BCL2 inhibitor, ABT-199

**Figure 4:**
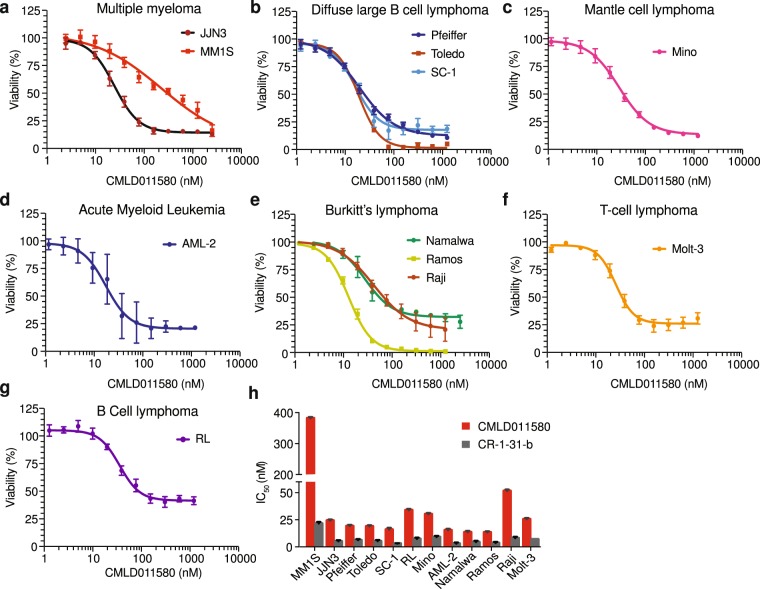
Dose-dependent inhibition of tumor cell survival in the presence of CMLD011580. (**a**–**g**) Tumor cells of different hematological origins [multiple myeloma (MM1S, JJN3), diffuse large B cell lymphoma (Pfeiffer, Toledo, SC-1), mantle cell lymphoma (Mino), acute myeloid leukemia (AML-2), Burkitt’s lymphoma (Namalwa, Ramos, Raji), T-cell lymphoma (Molt-3) and B-cell lymphoma (RL)] were incubated with the indicated concentrations of CMLD011580 for 48 hrs and cell viability assessed by CellTiter Glo. Results are expressed relative to vehicle controls and are the mean ± SEM of 3 biological replicates. (**h**) IC_50_ of (−)-CR-1-31-b and CMDL011580 towards the indicated cells lines. Results are expressed as mean ± SEM of 3 biological replicates.

Given these results towards murine B cell lymphomas, we assessed the cytotoxic activity of CMLD011580 against a number of human hematological cancer types that included multiple myeloma, diffuse large B cell lymphoma (DLBCL), mantle cell lymphoma (MCL), acute myeloid leukemia (AML), Burkitt’s lymphoma (BL), T-cell (TCL) and B-cell (BCL) lymphoma (Fig. 4a–h). In general, CMLD011580 was effective at inducing cell death in these lines - displaying an IC_50_ in the 10–50 nM range. These results indicate that CMLD011580 is effective against a set of cancer cell lines of different hematological origins.

**Figure 5:**
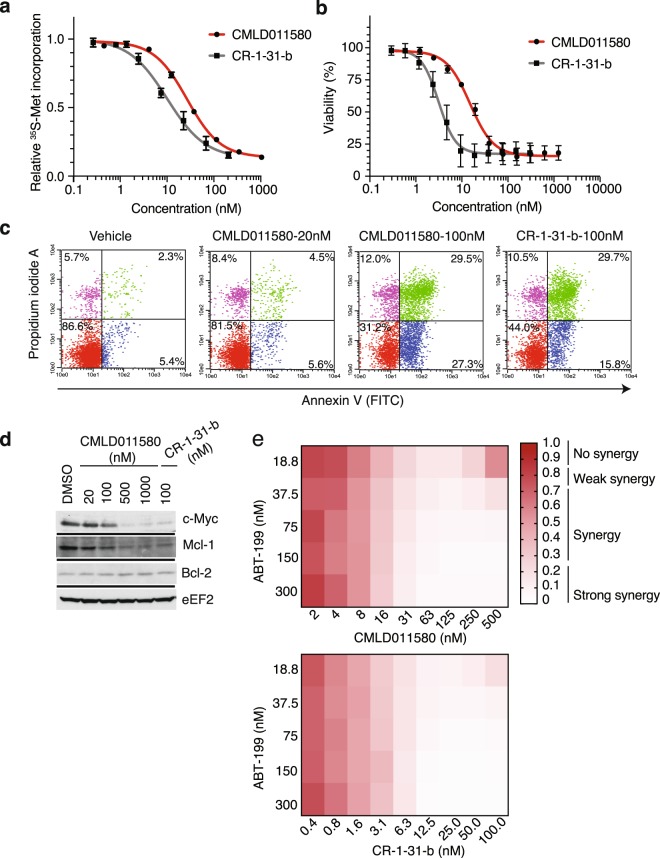
Biological properties of CMLD011580 on the THL-DLBCL cell line, SC-1. (**a**) Dose-dependent inhibition of protein synthesis by (−)-CR-1-31-b and CMLD011580 in SC-1 cells. Cells were incubated with the indicated concentrations of compound for 1 h and [^35^S]-methionine was added 15 minutes before the end of the incubation. The rate of [^35^S]-methionine incorporation is expressed relative to that of cells treated with vehicle (DMSO). Results are expressed as mean ± SEM of 3 biological replicates. (**b**) Relative viability of SC-1 cells exposed to (−)-CR-1-31-b or CMLD011580 for 48 h was assessed by CellTiter Glo. Results are expressed as mean ± SEM of 3 biological replicates. (**c**) CMLD011580 induces apoptosis in SC-1 cells. Apoptosis was assessed 48 h after compound addition at the indicated concentrations by annexin V and PI staining. Presented is a representative image of 2 independent experiments. (**d**) Western blot of extracts prepared from SC-1 cells treated with the indicated compounds concentrations for 5 h and probed with antibodies to the indicated proteins. Blots were obtained from the same membrane sequentially probed with the indicated antibodies and are cropped for ease of comparison. (**e**) Synergistic activity between ABT-199 and CMLD011580 or (−)-CR-1-31-b in SC-1 cells. Cell viability was measured 24 h following incubation with combinations of either of the compounds with ABT-199. n = 3.

SC-1, a follicular non-Hodgkin lymphoma subtype harboring chromosomal rearrangements in MYC, BCL6 and BCL2, was among the cell lines found to be most sensitive to (−)-CR-1-31-b and CMLD011580 (Fig. 4b). Such lymphomas are characterized as triple-hit lymphomas (THL) and exhibit highly aggressive clinical behaviour and poor prognosis^27^. We found that (−)-CR-1-31-b and CMLD011580 potently inhibited protein synthesis in SC-1 cells at IC_50_s of 15 and 30 nM respectively (Fig. 5a). This activity translated into high cytotoxicity in a dose-dependent manner through induction of a strong apoptotic response (Fig. 5b,c). The synthesis of the short-lived c-MYC and MCL-1 proteins were curtailed following a 5 h exposure of SC-1 cells to both compounds, whereas levels of the longer lived Bcl-2 protein was not affected during this acute treatment (Fig. 5d). These results lead us to test whether CMLD011580 could synergize with the potent and selective BCL-2 inhibitor, ABT-199. Indeed, when combining either CMLD011580 or (−)-CR-1-31-b with ABT-199, we observed a synergistic effect in the drug combinations at concentrations as low as 16 and 6.3 nM for CMLD011580 and CR-1-31-b, respectively (Fig. 5e).

### CMLD011580 synergizes with dexamethasone in multiple myeloma

**Figure 6:**
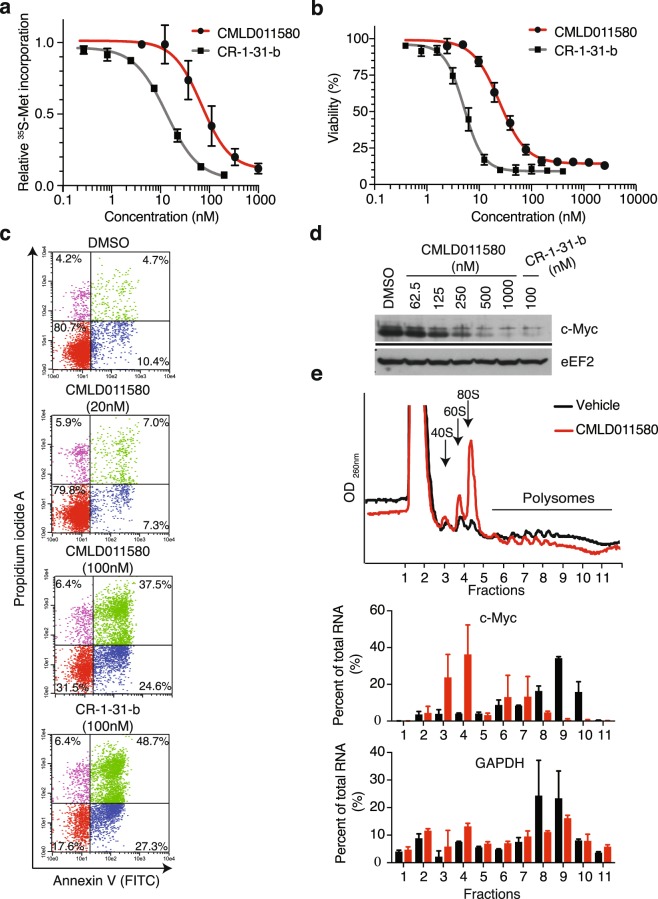
Effect of CMLD011580 on multiple myeloma JJN3 cell viability. (**a**) Dose-dependent inhibition of protein synthesis by (−)-CR-1-31-b and CMLD011580 in JJN3 cells. Cells were incubated with the indicated compound concentrations for 1 h and [^35^S]-methionine was added 15 minutes before the end of the incubation. The rate of [^35^S]-methionine incorporation is expressed relative to that of cells treated with vehicle (DMSO). Results are expressed as mean ± SEM of 3 biological replicates. (**b**) Relative survival of JJN3 cells exposed to (−)-CR-1-31-b or CMLD011580 for 48 h with cell viability assessed by CellTiter Glo. Results are expressed as mean ± SEM of 3 biological replicates. (**c**) CMLD011580 induces apoptosis in JJN3 cells. Apoptosis was assessed 24 h after compound addition at the indicated concentrations by annexin V and propidium iodide staining. Presented is a representative image of 2 experiments. (**d**) Western blot of extracts prepared from JJN3 cells treated with the indicated compound concentrations (nM) for 5 h and probed with antibodies to the proteins indicated to the right. Blots were obtained from the same membrane sequentially probed with the indicated antibodies and are cropped for ease of comparison. (**e**) Polysome analysis of JJN3 cells treated with vehicle (0.05% DMSO) or 25 nM CMLD011580 for 1 h. Polysomes were fractionated on a 10–50% sucrose gradient. The distribution of MYC and GAPDH mRNA within the polysome fractions of vehicle (black boxes) or CMLD011580 (red boxes) treated cells was measured by RT-qPCR. Results are expressed as mean ± SEM of 2 biological replicates.

We previously found that multiple myeloma cells are quite sensitive to rocaglates^28^. We thus assessed the activity of CMLD011580 against the multiple myeloma cell line JJN3. This compound caused a rapid depletion of protein synthesis as observed by ^35^S-methionine incorporation, with an IC_50_ of ~90 nM after 1 h exposure (Fig. 6a). (−)-CR-1-31-b proved to be more potent with an IC_50_ of ~15 nM (Fig. 6a). The observed inhibition of translation was not a consequence of eIF2α phosphorylation (Suppl. Fig. 4). Both compounds were cytotoxic against JJN3 cells (Fig. 6b; CMLD011580 IC_50_ = ~30 nM, (−)-CR-1-31-b IC_50_ = 5 nM) and induced a potent apoptotic response in cells as assessed by annexin V and PI staining (Fig. 6c). As previously reported for rocaglates, CMLD011580 inhibited MYC mRNA translation (Fig. 6d,e)^5,28^.

**Figure 7:**
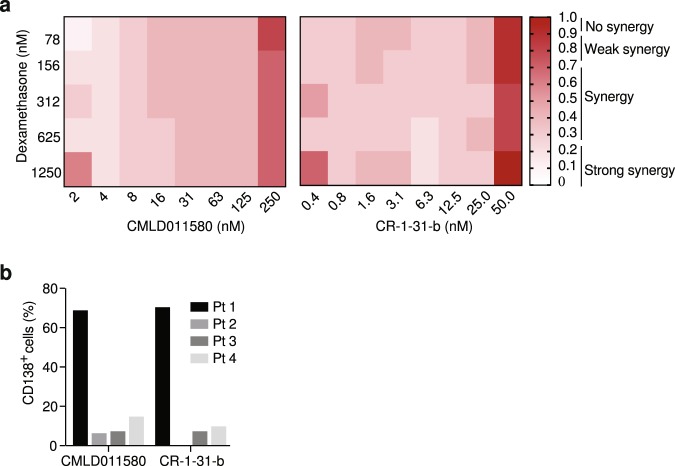
Biological activity of CMLD011580 in multiple myeloma cells. (**a**) DEX-dependent synergy with CMLD011580 in JJN3 cells. Cell viability was measured with CellTiter Glo after 48 hours of incubation with different drug combinations. n = 3. (**b**) Primary human MM cells are sensitive to CMLD011580. Primary tumor cells were exposed *ex vivo* to 50 nM CMLD011580 or (−)-CR-1-31-b for 72 h, after which point cell viability was determined by flow cytometry as indicated in the Materials and Methods. Presented is the percentage of CD138^+^ cells surviving drug exposure relative to DMSO controls.

We previously found that the rocaglate silvestrol could synergize with dexamethasone (DEX) to induce cell death in multiple myeloma cells^28^ and therefore assessed if CMLD011580 would show a similar behaviour (Fig. 7a). A series of extended titrations were performed with CMLD011580 and DEX on dexamethasone-resistant JJN3 cells^28^ and revealed significant synergy (combination index below 0.25) for the DEX/CMLD011580 combination at concentrations that extended as low as 2 nM and over a 2-log_10_ concentration range (Fig. 7a). (−)-CR-1-31-b also displayed synergy against JJN-3 cells and this effect extended to 0.4 nM (Fig. 7a). We also assessed CMLD011580’s cytotoxic activity against primary multiple myeloma patient samples and observed significant depletion of CD138^+^ tumor cells following 72 h exposure to CMLD011580 or (−)-CR-1-31-b in 3 out of 4 tested samples (Fig. 7b). These results indicate that CMLD011580 is effective as a single agent towards multiple myeloma cells and can synergize with dexamethasone.

## Discussion

Herein, we compare the translation inhibitory activity of two aglaiastatin analogues (CMLD010582 and CMLD011580) as well as aglaroxin C (CMLD10833) to one of the more potent rocaglate hydroxamates, (−)-CR-1-31-b (Fig. 1a). CMLD010582 showed no activity at inhibiting *in vitro* translation in comparison to the natural product, aglaroxin C (CMLD010833) (Fig. 1b), likely due to the additional angular methyl group present in the derivative CMLD010582. *In vitro*, the synthetic derivative *oxo*-aglaiastatin CMLD011580 was more potent than CMLD010582 and CMLD010833 (Fig. 1b), while *in vitro* and *ex vivo* CMLD011580 showed a ~10–20 fold decreased potency relative to (−)-CR-1-31-b at inhibiting translation and inducing cell death (Figs 1, 5 and 6). The biological properties of CMLD011580, like that of rocaglates, appear to be primarily a consequence of targeting eIF4A1 (Fig. 2). CMLD011580 and CR-1-31-b causes clamping of eIF4A1 onto RNA enriched for poly (AG)_8_ sequences (Fig. 2c), a feature also shown for RocA and which could result in inhibition of initiation by interfering with scanning ribosomes^8^. Clearly not all mRNAs are substrates for this clamping-mediated inhibition, since compounds did not stimulate binding to poly (UC)_8_ and we observed stimulation of unwinding of a dsRNA duplex with a poly(A) rich single-stranded 5′ end extension (Suppl. Fig. 2e)^5^. CMLD011580 induced cell death in a number of cell lines of different hematological origins (Fig. 4), *ex vivo* showed synergy with the glucocorticoid dexamethasone against multiple myeloma (JJN3) cells and with ABT-199 towards the THL SC-1 cell line, and *in vivo* synergized with doxorubicin against *Myr-Akt/Eμ-Myc* lymphomas (Figs 3, 5 and 7). *In vivo* CMLD011580 appeared to inhibit translation in livers of treated mice for a much shorter time period than (−)-CR-1-31-b (Fig. 3a; 1 h versus 3 h). These results indicate that although less potent and less stable *in vivo*, CMLD011580 functions analogously to CR-1-31-b.

CMLD011580 has previously been shown to inhibit gene expression in cell lines expressing either a CMV-driven or HSF1-dependent reporter^21^. However, these cell-based assays did not determine which step of gene expression was being targeted. As well, overnight compound exposure was used to assess effects on gene expression, making it difficult to draw conclusions concerning the potency or selectivity towards the translation apparatus from this earlier study. The fact that eIF4A1^em1JP^ cells, which are derived from NIH/3T3 cells and harbor eIF4A1 alleles containing a F163L mutation which imparts resistance to rocaglates, are also resistant to CMLD011580 (Fig. 2a) indicates that CMLD011580 targets translation through inhibition of eIF4A and is not a pan-DEAD box helicase inhibitor.

CMLD011580 was effective against a number of hematological cell lines with various mutational landscapes. We note that among the cell lines tested, MM1S were the least sensitive to the cytotoxic activity of both CMLD011580 and (−)-CR-1-31-b (Fig. 4h). Although, we do not understand the molecular basis for this, it is worth mentioning that a single missense mutation in eIF4A1 can lead to rocaglate resistance^5^. It remains to be established if a similar mutation could be responsible for the reduced sensitivity of these cells to CMLD011580 (and (−)-CR-1-31-b). Moreover, the naturally occurring rocaglate silvestrol is a known target of Pgp-1, the product of the multidrug-resistant gene, isoform1 (ABCB1). However, this does not appear to be a property of CMLD011580, as assessed in Hela cells overexpressing Pgp-1 (Suppl. Fig. 3). We also do not know if CMLD011580 accumulates to the same levels in the different cell lines studied herein.

We have previously shown that targeting eIF4A with hippuristanol, a small molecule that inhibits eIF4A RNA binding, sensitizes MYC-driven tumors to Bcl-2 targeted therapies^29^. In that study, combining hippuristanol with ABT-737 synergized against Eμ-Myc-driven lymphomas, as well as a number of human lymphoma cell lines. ABT-737 is a Bcl-2 Homology 3 (BH3)-mimetic that induces apoptosis by binding to Bcl-2, Bcl-xL, and Bcl-w, and in the aforementioned study we did not determine if the results were the consequence of inhibiting one specific Bcl-2 family member, some, or all three. Here we demonstrate that targeting Bcl-2 with the more selective inhibitor, ABT-199, is sufficient to synergize with eIF4A inhibition mediated by CMLD011580 in the triple-hit lymphoma cell line, SC-1. Triple hit lymphomas harbor MYC, BCL-2, and BCL-6 rearrangements and have an aggressive clinical course and poor prognosis. The ability of ABT-199 to antagonize BCL-2 and of CMLD011580 to inhibit MYC expression may account for the synergy seen with these two compounds. Additionally, since upregulation of MCL-1 (and BFL-1) can lead to acquired resistance to BCL-2 antagonists^30^, an added benefit of CMDL011580 (and CR-1-31-b) is blockade of MCL-1 translation thus curtailing this particular resistance mechanism.

We also find that CMLD011580 can synergize with the DNA damaging agent, doxorubicin against *Myr-Akt/Eμ-Myc* lymphomas *in vivo* (Fig. 3b), despite the apparent inhibition of translation exerted by CMDL011580 *in vivo* being significantly shorter than (−)-CR-1-31-b (Fig. 3a). These results are consistent with the notion that transient suppression of protein synthesis is sufficient to blunt key cancer dependency networks, among which are pro-survival pathways which enable DXR to trigger cell death. In sum, CMLD011580 targets translation through modulation of eIF4A activity, shows single agent activity *in vitro*, and can reverse drug resistance *in vivo*. Future studies will aim to modify the *oxo*-aglaiastatin core to improve potency as a translation inhibitor.

## Methods

### Chemical reagents

The rocaglate (−)-CR-1-31-b was synthesized as previously described^20^. (−)-Aglaroxin C (CMLD010833), (+)-*oxo*-aglaiastatin (CMLD011580), and the aglaiastatin analog (±)-CMLD010582 were synthesized according to literature procedures^21^. For synthesis and characterization of CMLD010582 and the enantioenriched CMLD011580, see Supplemental Methods. Dexamethasone and ABT-199 were obtained from Sigma and SelleckChem, respectively. All compounds were resuspended in DMSO and stored at −80 °C.

### Cell lines and primary cells

HEK293T/17 and NIH3T3 were cultured in DMEM supplemented with 10% (v/v) FBS, 100 U/ml penicillin/streptomycin and 2 mM of *L*-glutamine. JJN3, MM1S, Pfeiffer, Toledo, SC-1, RL, Mino, AML-2, Namalwa, Ramos, Raji and Molt-3 were maintained in RPMI supplemented with 10% (v/v) FBS, 100U/ml penicillin/streptomycin and 2 mM glutamine. Multiple myeloma patient bone marrow samples were recovered according to a McGill University Health Centre Institutional Review Board-approved informed consent protocol. All methods were performed in accordance with the relevant guidelines and regulations. Mononuclear cells were recovered and maintained in IMDM supplemented with 15% FBS in the presence of vehicle (DMSO) or the compounds of interest for a period of 72 hours. Apoptosis was measured by flow cytometry using a CD138-V450 (BD Biosciences) and Annexin V-APC (BD Biosciences) antibodies and stained samples were analyzed using a FACSCanto II instrument (BD Biosciences). The data was further analyzed using FlowJo (FlowJo LLC) and FCS Express 4 (De Novo Software).

### Immunoblotting

All extracts were prepared in RIPA lysis buffer (20 mM Tris [pH 7.6], 100 mM NaCl, 1 mM EDTA, 1 mM EGTA, 1% NP40, 0.5% sodium deoxycholate, 0.1% SDS, 1 mM PMSF, 4 μg/ml aprotinin, 2 μg/ml leupeptin, 2 μg/ml pepstatin) and resolved on an 8% (to detect Pgp-1) or 10% SDS/PAGE (for all other proteins) before transfer on a PVDF membrane (Bio-Rad). The following antibodies were used for protein expression detection: p-eIF2α, eIF2α, eEF2, Bcl-2, Mcl-1, GAPDH (Cell Signaling), c-Myc (N262, Santa Cruz), and Pgp-1 (Abcam).

### Polysome profiles and quantitative real-time PCR

For polysome profiling studies, lymphoma cells were treated with vehicle (DMSO) or drugs of interest for 1 h before being lysed in the presence of 100 μg/ml cycloheximide in a hypotonic lysis buffer containing 5 mM Tris (pH 7.5), 2.5 mM MgCl_2_, 1.5 mM KCl, 2 mM DTT, 1% Triton X-100 and 0.5% sodium deoxycholate. The lysates were then loaded on a 10–50% sucrose gradient and centrifuged for 2.25 h at 35000 rpm and 4 °C. The absorbance was measured and fractions collected using the FOXY R1 (ISCO) fraction collector. RNA from the collected fractions was isolated using TRIzol (Life Technologies) according to the manufacturer’s instructions. Myc and GAPDH mRNA levels were assessed by RT-qPCR using the CFX96 PCR System (Bio-Rad) and the following primers: *gapdh* forward 5′-GAAGGTGAAGGTCGGAGTC-3′, *gapdh* reverse 5′-GAAGATGGTGATGGGATTC-3′; *myc* forward 5′- CACCACCAGCAGCGACTCT-3′, *myc* reverse 5′-CTGACCTTTTGCCAGGAGC-3′.

### Cell viability assay and median effect analysis

The relative cell growth was measured in 96-well plates using the luminescent detection of CellTiter Glo (Promega) following the manufacturer’s instructions. For NIH3T3, 5000 cells were plated per well and incubated in compound for 48 h. For the determination of IC_50_, all suspension cells were plated at 500,000 cells/ml and incubated with increasing drugs concentration for 48 h. All experiments were performed three times. IC_50_ values were calculated using a four-parameter non-linear curve-fit analysis using GraphPad Prism 7 software.

For synergy assays, 500,000 JJN3 or SC-1 cells were seeded in 96-well plates in the presence of increasing concentrations of drugs. Twenty-four to forty-eight hours later, viability was assessed using CellTiter-Glo. Values obtained were standardized against vehicle (DMSO) control, which was set at 1. To characterize synergy, data were analyzed using the median effect method and CompuSyn^31^. CI values < CI < 0.75 indicates moderate synergy, 0.75 < CI < 1.0 indicates weak synergy, and CI > 1.0 indicates antagonism.

### *In vivo* metabolic labeling studies

Assessment of compound activity on DNA transcription and protein synthesis were performed as follows: Twenty-four hours prior to the experiment, 6 × 10^4^ Hela cells were plated in a 24-well plate. Cells were treated with either (−)-CR-1-31-b or CMLD011580 for 45 min before ^35^[S]-methionine (150–225 μCi/ml) or ^3^[H]-uridine (24 μCi/ml) were added for 10 minutes, after which cells were harvested.^3^[H]-uridine was added to cells in DMEM supplemented with 10% dialyzed FCS. ^35^[S]-methionine was added to cells in methionine-free media supplemented with 10% dialyzed FCS. Cells were harvested and lysed in RIPA buffer. Radiolabelled proteins were precipitated on Whatman paper and radiolabelled nucleic acids were extracted by filtration through Whatman GF/C glass fiber filters. Assessment of radioactivity was determined by scintillation counting. All counts were then normalized to protein concentration, determined with a DC Protein Assay (Bio-Rad)

### *In vitro* translation assays

*In vitro* translation assays were performed in Krebs-2 cell extracts at a final mRNA concentration of 4 μg/ml. The firefly and Renilla luciferase activities were measured on a Berthold Lumat LB 9507 luminometer (Berthold technologies). All *in vitro* transcriptions and translation assays were performed as previously described^32^.

### Fluorescence polarization assay

Purified eIF4A1 (100 nM) was incubated with 10 nM of 5′FAM-labelled RNA (IDT) in the presence of 0.5% DMSO or increasing concentrations of (−)-CR-1-31-b or CMLD011580. All experiments were performed in the presence of 1 mM of AMPPNP in a 10 ul reaction containing 14.4 mM HEPES-NaOH (pH 8), 108 mM NaCl, 1 mM MgCl_2_, 14.4% glycerol, and 2 mM DTT and were incubated for 30 min at room temperature prior to FP measurements (PHERAstar FS).

### Pharmacological studies

All pharmacological studies were performed by Cyprotex (United States) following their standard protocols. Plasma stability was assessed using propantheline and warfarin as controls for low and high plasma stability respectively. Briefly, 5 μM of CMLD011580 was incubated in plasma at 37 °C. At indicated time points (five time-points to determine half-life), an aliquot of the mixture was removed, deproteinated and the samples analyzed by liquid chromatography/mass spectrometry (LC-MS/MS).

The ability of the drug to bind plasma protein was measured by incubating the compound in plasma in a Rapid Equilibrium Dialysis device and dialyzing against PBS. Four hours later, each side was analyzed by LC/MS/MS. Results are presented as the percent of protein bound and unbound.

Microsomal clearance was determined by incubating 1μM of compound in microsomes in the presence of NADPH at 37 °C. At indicated time points (five time-points to determine half-life), an aliquot of the mixture is removed, deproteinated and extracts analyzed by LC-MS/MS.

### Animal Studies

All animal studies performed were approved by the McGill University Faculty of Medicine Animal Care Committee. All methods were performed in accordance with the relevant guidelines and regulations of the Canadian Council on Animal Care. Liver polysome profiles were assessed in 8–10- weeks old C57BL/6 mice. Animals were injected intraperitoneally with vehicle or 0.2 mg/kg of (−)-CR-1-31-b or CMLD011580. Livers were collected 1 or 3 hours later and washed in cold PBS containing 100μg/ml cycloheximide. Homogenization and sample preparation was performed as previously described^29^.

For *in vivo* chemosensitization assays, 9 -weeks old female C57BL/6 mice were injected by tail vein with 1 × 10^6^ *Myr-Akt/Eμ-Myc* lymphoma cells per mouse. Following the development of palpable tumors in mice inguinal lymph nodes, mice were treated by intraperitoneal injection for 5 consecutive days with (−)-CR-1-31-b or CMLD011580 (0.2 mg/kg) and/or doxorubicin (10 mg/kg) once on day 2. The latter was prepared in ddH_2_O while rocaglamide derivatives were administered in a 5.2% PEG400, 5.2% Tween-80. Tumor-free survival was assessed by measuring the time between complete tumor regression following drug administration and the reappearance of tumors in the inguinal lymph nodes.

## Supplementary information

Supplementary Information

## References

[1] Gingras AC, Raught B, Sonenberg N (1999). eIF4 initiation factors: effectors of mRNA recruitment to ribosomes and regulators of translation. Annu Rev Biochem.

[2] Sonenberg N, Hinnebusch AG (2009). Regulation of translation initiation in eukaryotes: mechanisms and biological targets. Cell.

[3] Bhat M (2015). Targeting the translation machinery in cancer. Nat Rev Drug Discov.

[4] Sadlish H (2013). Evidence for a functionally relevant rocaglamide binding site on the eIF4A-RNA complex. ACS Chem Biol.

[5] Chu J (2016). CRISPR-Mediated Drug-Target Validation Reveals Selective Pharmacological Inhibition of the RNA Helicase, eIF4A. Cell Rep.

[6] Bordeleau ME (2008). Therapeutic suppression of translation initiation modulates chemosensitivity in a mouse lymphoma model. J Clin Invest.

[7] Cencic R (2009). Antitumor activity and mechanism of action of the cyclopenta[b]benzofuran, silvestrol. PLoS One.

[8] Iwasaki S, Floor SN, Ingolia NT (2016). Rocaglates convert DEAD-box protein eIF4A into a sequence-selective translational repressor. Nature.

[9] Hwang BY (2004). Silvestrol and episilvestrol, potential anticancer rocaglate derivatives from Aglaia silvestris. J Org Chem.

[10] Lucas DM (2009). The novel plant-derived agent silvestrol has B-cell selective activity in chronic lymphocytic leukemia and acute lymphoblastic leukemia *in vitro* and *in vivo*. Blood.

[11] Santagata S (2013). Tight coordination of protein translation and HSF1 activation supports the anabolic malignant state. Science.

[12] Rubio CA (2014). Transcriptome-wide characterization of the eIF4A signature highlights plasticity in translation regulation. Genome Biol.

[13] Wolfe AL (2014). RNA G-quadruplexes cause eIF4A-dependent oncogene translation in cancer. Nature.

[14] King ML (1982). X-Ray Crystal Structure of Rocaglamide, a Novel Antileukemic I H-Cyclopenta[b] benzofuran from Aglaia elliptifolia. J. Chem. Sco. Chem. Commun..

[15] Proksch P, Edrada RA, Ebel R, Bohnenstengel FI, Nugroho BW (2001). Chemistry and Biological Activity of Rocaglamide Derivatives and Related Compounds in Aglaia Species (Meliaceae). Curr. Org. Chem..

[16] Kim S, Salim AA, Swanson SM, Kinghorn AD (2006). Potential of cyclopenta[b]benzofurans from Aglaia species in cancer chemotherapy. Anticancer Agents Med Chem.

[17] Ebada SS, Lajkiewicz N, Porco JA, Li-Weber M, Proksch P (2011). Chemistry and biology of rocaglamides (=flavaglines) and related derivatives from aglaia species (meliaceae). Prog Chem Org Nat Prod.

[18] Wang W, Cencic R, Whitesell L, Pelletier J, Porco JA (2016). Synthesis of Aza-Rocaglates via ESIPT-Mediated (3 + 2) Photocycloaddition. Chemistry.

[19] Gerard B, Cencic R, Pelletier J, Porco JA (2007). Enantioselective synthesis of the complex rocaglate (−)-silvestrol. Angew Chem Int Ed Engl.

[20] Rodrigo CM, Cencic R, Roche SP, Pelletier J, Porco JA (2012). Synthesis of rocaglamide hydroxamates and related compounds as eukaryotic translation inhibitors: synthetic and biological studies. J Med Chem.

[21] Stone SD, Lajkiewicz NJ, Whitesell L, Hilmy A, Porco JA (2015). Biomimetic kinetic resolution: highly enantio- and diastereoselective transfer hydrogenation of aglain ketones to access flavagline natural products. J Am Chem Soc.

[22] An FL (2016). Cytotoxic Rocaglate Derivatives from Leaves of Aglaia perviridis. Sci Rep.

[23] Mi, Q. *et al*. Silvestrol regulates G2/M checkpoint genes independent of p53 activity. *Anticancer Res* **26**, 3349-3356 (2006). 17094452

[24] Adams JM (1985). The c-myc oncogene driven by immunoglobulin enhancers induces lymphoid malignancy in transgenic mice. Nature.

[25] Schmitt CA, Rosenthal CT, Lowe SW (2000). Genetic analysis of chemoresistance in primary murine lymphomas. Nat Med.

[26] Wendel HG (2004). Survival signalling by Akt and eIF4E in oncogenesis and cancer therapy. Nature.

[27] Wang W, Hu S, Lu X, Young KH, Medeiros LJ (2015). Triple-hit B-cell Lymphoma With MYC, BCL2, and BCL6 Translocations/Rearrangements: Clinicopathologic Features of 11 Cases. Am J Surg Pathol.

[28] Robert F (2014). Translation initiation factor eIF4F modifies the dexamethasone response in multiple myeloma. Proc Natl Acad Sci USA.

[29] Cencic R (2013). Modifying chemotherapy response by targeted inhibition of eukaryotic initiation factor 4A. Blood Cancer J.

[30] Yecies D, Carlson NE, Deng J, Letai A (2010). Acquired resistance to ABT-737 in lymphoma cells that up-regulate MCL-1 and BFL-1. Blood.

[31] Chou T, Talalay P (1984). Quantitative analysis of dose-effect relationships: the combined effects of multiple durgs or enzyme inhibitors. Adv. Enzyme Regul..

[32] Novac O, Guenier AS, Pelletier J (2004). Inhibitors of protein synthesis identified by a high throughput multiplexed translation screen. Nucleic Acids Res.

