# Supplementary material for: Oxo-aglaiastatin-Mediated Inhibition of Translation Initiation
Source: Sci Rep. 2019 Feb 4;9:1265. doi: 10.1038/s41598-018-37666-5 (PMC6361980; doi:10.1038/s41598-018-37666-5)
Supplement: Supplementary file 1 — Supplementary Information [file 41598_2018_37666_MOESM1_ESM.pdf]

Oxo-aglaiastatin-Mediated Inhibition of Translation Initiation

Rayelle Itoua Maïga[1], Regina Cencic[1], Jennifer Chu[1], Daniel D. Waller[3], Lauren E. Brown[2], William G. Devine[2], Wenhan Zhang[2], Michael Sebag[3], John A. Porco, Jr.[2], Jerry Pelletier[1,4,5]

[1]Department of Biochemistry, McGill University, [2]Department of Chemistry and Center for Molecular Discovery (BU-CMD), Boston University, [3]Department of Medicine, [4]Department of Oncology, [5]Rosalind & Morris Goodman Cancer Research Centre, McGill University, Montreal, Québec, Canada, H3G 1Y6

SUPPLEMENTAL METHODS

Synthesis scheme and characterization of CMLD010582

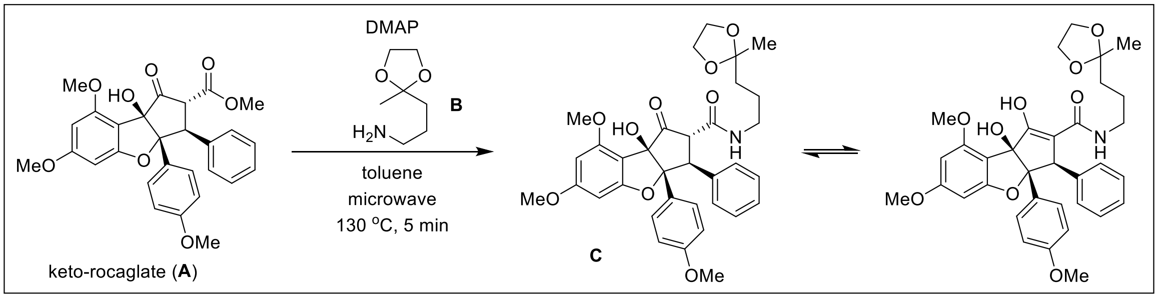

(2*R*,3*S*,3a*R*,8b*R*)- 8b-Hydroxy-6,8-dimethoxy-3a-(4-methoxyphenyl)-*N*-(3-(2-methyl-1,3-dioxolan-2-yl)propyl)-1-oxo-3-phenyl-2,3,3a,8b-tetrahydro-1*H*-cyclopenta[*b*]benzofuran-2-carboxamide C: To a solution of keto-rocaglate **A** (200 mg, 0.41 mmol)[1] in dry toluene (4.0 mL) was added 3-(2-methyl-1,3-dioxolan-2-yl)propan-1-amine **B** (71 mg, 0.49 mmol) and 4-dimethylaminopyridine (12 mg, 98 µmol). The mixture was irradiated to 130 °C in a microwave reactor where the temperature was held for 5 minutes. The mixture was concentrated *in vacuo* and used without further purification. Alternatively, the crude amide product **C** could be purified for analysis by flash column chromatography on SiO_2 using a gradient of 50-80% EtOAc/hexanes. **TLC:** (70% EtOAc/hexanes) R_f = 0.26, fluoresces yellow under long wave UV; **m.p.** 91-95 °C; **IR:** (diamond-ATR, cm^{-1}) 3370 (br.), 2938, 1737, 1658, 1604, 1251, 1147, 1034, 697; **^{1}H NMR** (400 MHz, $CDCl_3$) δ ppm 7.01 - 7.12 (m, 8H), 6.85 - 7.01 (m, 6H), 6.65 (d, *J* = 9.0 Hz, 2H), 6.58 (t, *J* = 5.5 Hz, 1H), 6.53 (d, *J* = 9.0 Hz, 2H), 6.32 (d, *J* =2 .0 Hz, 1 H), 6.20 (d, *J* = 2.0 Hz, 1H), 6.05 (t, *J* = 2.1 Hz, 2H), 4.97 (t, *J* = 5.7 Hz, 1H), 4.31 (d, *J* = 12.9 Hz, 1H), 4.26 (s, 1H), 3.83 - 3.89 (m, 5H), 3.79 - 3.83 (m, 8H), 3.78 (s, 4H), 3.75 (s, 3H), 3.69 - 3.74 (m, 2H), 3.67 (s, 3H), 3.63 (s, 3H), 3.10 - 3.27 (m, 4H), 2.95 - 3.06 (m, 1H), 1.44 - 1.62 (m, 4H), 1.24 - 1.37 (m, 5H), 1.22 (s, 3H), 1.11 (s, 3H); **^{13}C NMR** (101 MHz, $CDCl_3$) δ ppm 207.1, 171.1, 169.0, 165.0, 164.2, 163.6, 161.3, 160.6, 158.8, 158.5, 158.5, 158.1, 137.0, 136.9, 128.9, 128.1, 128.0, 127.9, 127.5, 126.8, 126.8, 126.0, 113.2, 112.1, 109.8, 109.5, 106.7, 106.0, 101.7, 101.6, 99.6, 93.0, 92.5, 90.0, 89.0, 88.7, 88.3, 64.6, 64.6, 64.5, 64.5, 57.2, 56.8, 55.8, 55.7, 55.6, 55.1, 55.0, 51.2, 39.7, 38.7, 35.8, 35.7, 23.9, 23.8, 23.7, 23.6; **HR/MS:** *m/z* Calcd. for $[C_{34}H_{37}NO_9+Na]^+$ 626.2366, found 626.2365 (-0.2 ppm).

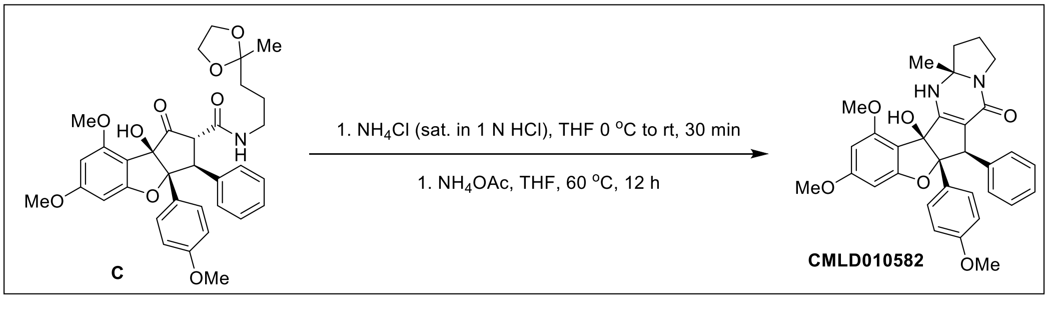

(6*R*,6a*R*,11b*S*,12a*S*)-11b-Hydroxy-9,11-dimethoxy-6a-(4-methoxyphenyl)-12a-methyl-6-phenyl-1,2,3,6,6a,11b,12,12a-octahydro-5*H*-benzofuro[2',3':4,5]cyclopenta[1,2-*d*]pyrrolo[1,2-*a*]pyrimidin-5-one (CMLD010582): Crude carboxamide **C** (123 mg, 0.20 mmol) was dissolved in THF (2.0 mL) and cooled to 0 °C. A saturated NH_4Cl/1M aq. HCl solution (2.0 mL) was added

dropwise. A precipitate formed immediately. After addition, the reaction was brought to room temperature and stirred for 30 minutes. The mixture was then extracted with ethyl acetate (3 x 20 mL). The combined organic layers were washed with brine (20 mL), dried over Na_2SO_4, filtered, and concentrated *in vacuo*. The crude material was taken forward without further purification. The crude hemiaminal (*not shown*) was dissolved in dry THF (6.4 mL) in a flame dried flask under argon. Ammonium acetate, dried with a vacuum dessicator for 4 h (157 mg, 2.0 mmol) was added and the mixture was heated to 60 °C for 12 h. The mixture was cooled to room temperature and ethyl acetate (20 mL) and water (20 mL) were added. The layers were separated and the aqueous was extracted with ethyl acetate (3 x 20 mL). The combined organic layers were washed with brine (20 mL), dried over Na_2SO_4, filtered, and concentrated *in vacuo*. The residue was purified by flash column chromatography on SiO_2 using a gradient of 40-100% EtOAc/hexanes to afford *C*-methyl-aglaiastatin (**CMLD010582**) as a white solid in 62% yield (68 mg, 0.13 mmol). **TLC:** (80% EtOAc/hexanes) R_f = 0.18, (EtOAc) R_f = 0.33, fluoresces bright blue under short wave UV; **m.p.** decomposed >180 °C; **IR:** (diamond-ATR, cm^{-1}) 3321 (br.), 2931, 1594, 1511, 1500, 1146, 1111, 808, 696, 540; **^{1}H NMR** (400 MHz, $CDCl_3$) δ ppm 7.11 (d, *J* = 8.6 Hz, 2H), 6.99 - 7.08 (m, 4H), 6.94 (t, *J* = 6.6 Hz, 1H), 6.59 (d, *J* = 8.6 Hz, 2H), 6.28 (s, 1H), 6.07 (s, 1H), 5.44 (s, 1H), 4.57 (s, 1H), 3.82 (s, 3H), 3.81 (s, 3H), 3.66 - 3.73 (m, 1H), 3.65 (s, 3H), 3.48 (dt, *J* = 11.9, 6.2 Hz, 1H), 2.25 (dt, *J* = 11.9, 6.2 Hz, 1H), 2.06 - 2.17 (m, 1H), 1.92 - 2.02 (m, 3H), 1.71 (s, 3 H); **^{13}C NMR** (101 MHz, $CDCl_3$) δ 164.0, 161.7, 161.2, 158.5, 157.5, 154.8, 139.2, 128.6, 128.2, 127.5, 127.4, 125.9, 112.6, 107.4, 105.3, 103.0, 92.9, 89.4, 88.1, 76.3, 57.6, 55.9, 55.6, 55.1, 44.6, 40.7, 25.0, 22.2; **HR/MS:** *m/z* Calcd. for $[C_{32}H_{32}N_2O_6+H]^+$ 541.2339, found 541.2333 (-1.1 ppm).

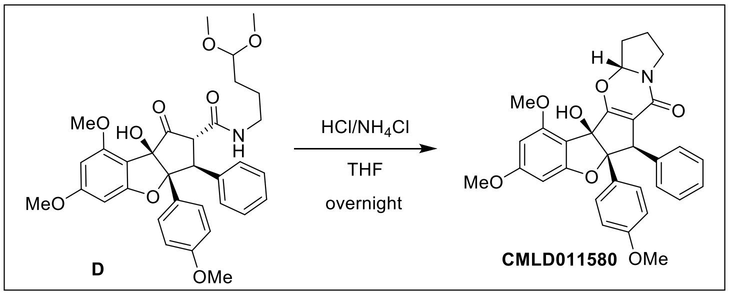

(6R,6aR,11bS,12aR)-11b-hydroxy-9,11-dimethoxy-6a-(4-methoxyphenyl)-6-phenyl-1,2,3,6a,11b,12a-hexahydrobenzofuro[3',2':3,4]cyclopenta[1,2-e]pyrrolo[2,1-b][1,3]oxazin-5(6H)-one (CMLD011580): Keto-rocaglamide **D** (43 mg, 0.072 mmol)[1] was dissolved in THF (0.72 ml, 0.1 M) and saturated NH_4Cl in 1 N HCl (0.72 ml, 0.1 M) was added. The mixture was stirred at room temperature overnight. The crude product was extracted three times using ethyl acetate, and the combined organic layer was washed by $NaHCO_3$ and brine followed by drying with Na_2SO_4. After concentration, the crude product was purified using preparative TLC (PTLC) (60% ethyl acetate in hexanes) to afford **CMLD011580** (9.5 mg, 22% yield). Characterization data matches our previous report.[1] $[\alpha]_D^{24}$ = + 4.0° (c = 0.25, $CHCl_3$)

Chiral HPLC analysis of (-)-**CMLD011580** and (+)-**CMLD011580**: A Chiralcel OD column was used with a 15% isopropanol in hexanes as the mobile phase at 1 mL/min flow rate.

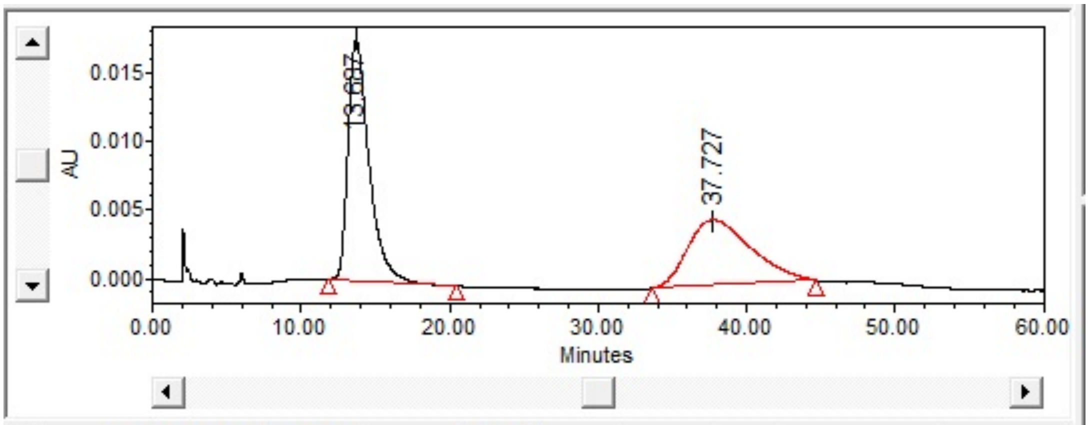

	Name	Retention Time (min)	Area (µV*sec)	% Area	Height (µV)	Int Type	Amount	Units	Peak Type	Peak Codes
1		13.687	1720695	54.88	17541	bb			Unknown	
2		37.727	1414828	45.12	4758	bb			Unknown	

Chiral HPLC analysis of (+)-CMLD011580: A Chiralcel OD column was used with a 15% isopropanol in hexanes as the mobile phase at 1 mL/min flow rate. 95% ee of (+)-**CMLD011580** was observed.

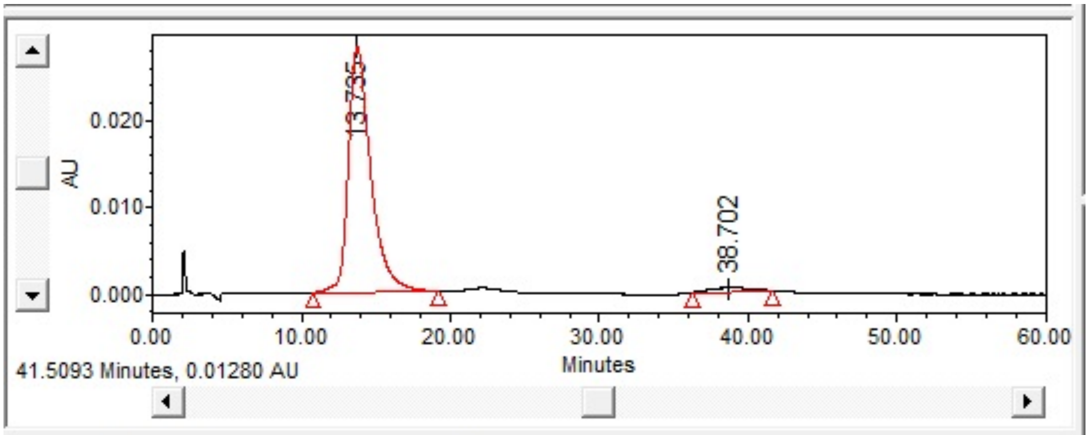

	Name	Retention Time (min)	Area (µV*sec)	% Area	Height (µV)	Int Type	Amount	Units	Peak Type	Peak Codes
1		13.735	2938486	97.18	28102	bb			Unknown	
2		38.702	85286	2.82	436	bb			Unknown	

[1] Stone, S. D.; Lajkiewicz, N. J.; Whitesell, L.; Hilmy, A.; Porco, J. A. *J. Am. Chem. Soc.* **2015**, *137*, 525.

SUPPLEMENTARY FIGURE LEGENDS

Supplementary Figure 1. Rocaglamide metabolites and derivatives. List of rocaglamides that have been tested as protein synthesis inhibitors.

Supplementary Figure 2. Activity Assessment of CMLD011580. **a.** Assessment of cap-dependent and HCV-mediated translation in the presence of the indicated compound concentrations in rabbit reticulocyte lysates. Translation extracts were programmed with FF/HCV/Ren mRNA (4 μg/ml). Luciferase activity (RLU) results are expressed relative to values obtained in the presence of DMSO controls. Results are expressed as mean ± SEM of 3 replicates. **b.** Assessment of cap-dependent translation in the presence of the indicated compound concentrations in wheat germ extracts. Translation extracts were programmed with FF/HCV/Ren mRNA (4 μg/ml). Firefly luciferase activity (RLU) results are expressed relative to values obtained in the presence of DMSO vehicle controls. Results are expressed as mean ± SEM of 3 replicates. Since the HCV IRES is not active in wheat germ extracts, renilla counts were not monitored. **c.** Effect of compounds on protein and RNA synthesis in HeLa cells. The rate of macromolecular synthesis (TCA precipitable cpm for a 10 min. labeling period) obtained in the presence of compound was normalized to the rate obtained in the presence of DMSO and is indicated. The results are shown for two experiments and the error of the mean is presented. The rate of protein and RNA synthesis in the vehicle control reactions (containing DMSO) averaged 238190 cpm/10 min, and 41920 cpm/10 min labeling, respectively. d. Cell cycle progression of Hela cells was assessed upon treatment of the cells with vehicle or the indicated drug concentrations for 24 h. Cells were then harvested, fixed and stained with propidium iodide. The results are presented as the relative percentage of cells in each cell cycle phase relative to the total population in G1, S or G2/M phases and are expressed as mean ± SEM of 3 biological replicates. **e.** Helicase activity of recombinant eIF4A1 (0.5 μg) and a radiolabeled RNA duplex5 in the presence of vehicle (DMSO), 10 or 50 μM of the indicated compound. RNA duplex was formed by annealing RNA-1 ($^{5'}$GGGGAGA(AAAAC)$_5$UAGCACCGUAAAGCACGA$^{3'}$) and ^{32}P-labelled RNA-11 ($^{5'}$GCUUUACGGUG$^{3'}$) and helicase assays were performed as previously described 5. n = 3-4 biological replicates ± SEM; * $p < 0.01$.

Supplementary Figure 3. CMLD011580 does not affect weight gain in mice and is not a substrate for Pgp-1. a. Long-term administration does not affect weight gain. Weight gain was monitored over a 7-week period after receiving 8 daily doses of 0.1 mg/kg CMLD011580 or 0.2 mg/kg

of CR-1-31-b. Weight variation refers to the difference in weight of mice relative to that on day 1, following the start of drug administration. Each cohort had 5 mice. Values are mean ± SEM. **b.** Titration of CMLD011580 and silvestrol on HeLa and HeLa/Pgp-1 cell lines. Cells were treated for 48 hrs with increasing concentrations of compound followed by assessment of cell proliferation using the CellTiter Glo. **c.** Western blot analysis of Pgp-1 in HeLa and HeLa/Pgp-1 cell lines.

Supplementary Figure 4. CMLD011580 does not induce eIF2α phosphorylation in JJN3 cells. Cells were treated with increasing doses of CMLD011580 or arsenite for 1.5 h. Cytoplasmic extracts were prepared, proteins resolved on 10% SDS-PAGE, and probed with antibodies to the indicated proteins.

Class	Cyclopenta[*bc*]benzopyrans	Benzo[*b*]oxepines	Cyclopenta[*b*] benzofurans (rocaglates)	Cyclopenta[*b*] benzofurans (Aglaiastatins)	Aza-rocaglates
**Structure**	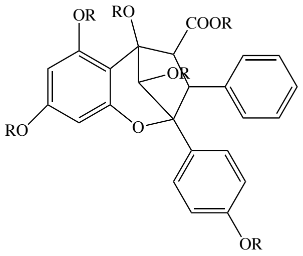	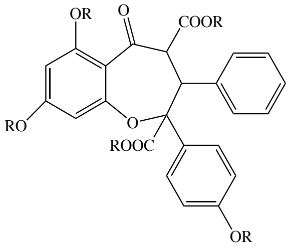	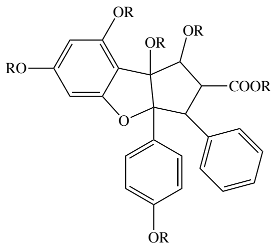	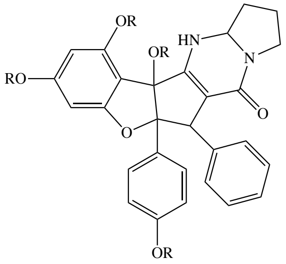	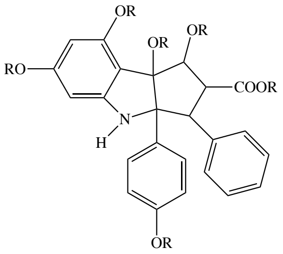
**Protein Synthesis Inhibition (Ref)**	Not Active [PMID 19401772]	Not Active [PMID 19401772]	Active [PMID 18551192]	Not Directly Tested	Not Active [PMID 27338157]

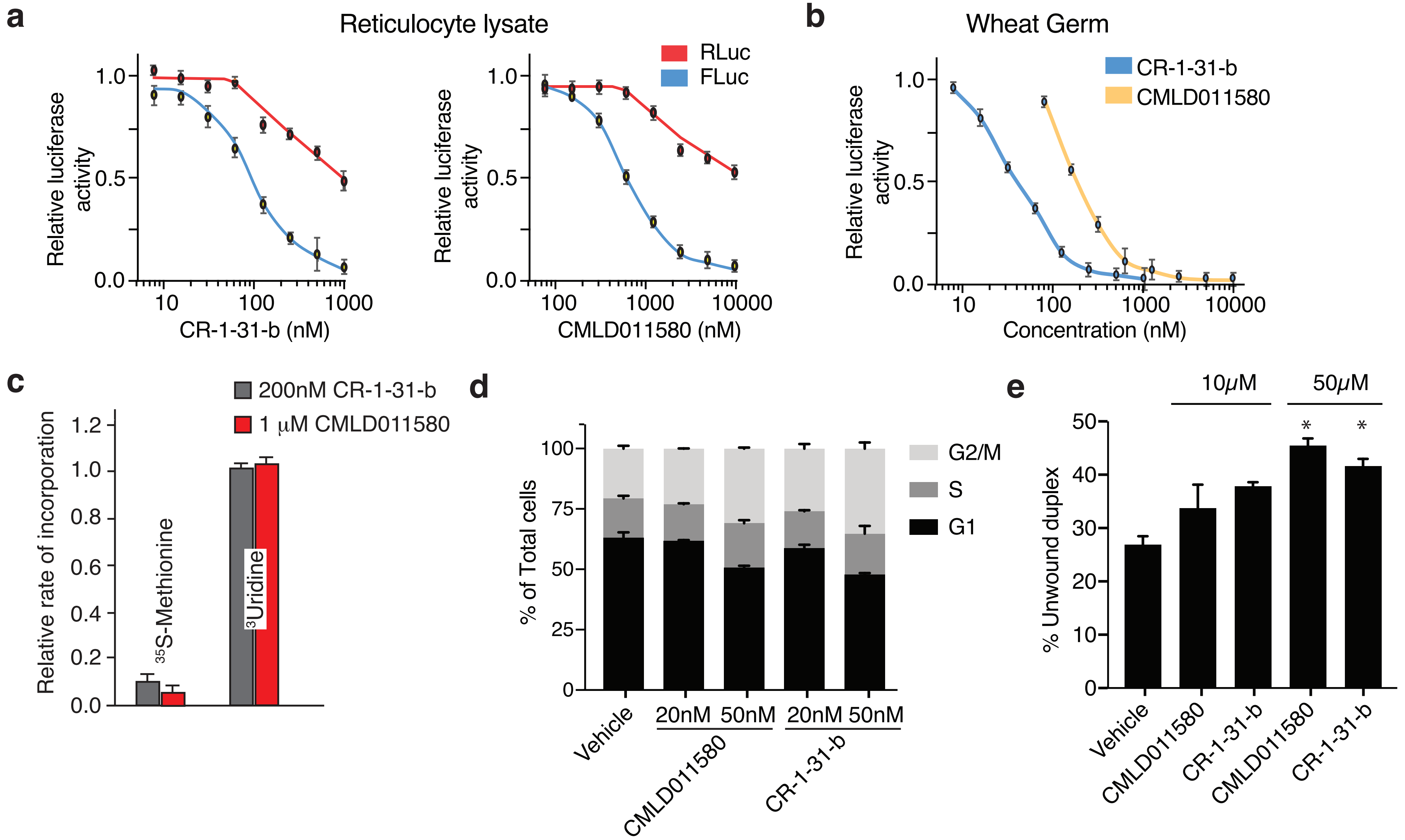

a
Reticulocyte lysate
RLuc
FLuc
Relative luciferase activity
CR-1-31-b (nM)
CMLD011580 (nM)
b
Wheat Germ
CR-1-31-b
CMLD011580
Relative luciferase activity
Concentration (nM)
c
200nM CR-1-31-b
1 μM CMLD011580
Relative rate of incorporation
35S-Methionine
3Uridine
d
% of Total cells
G2/M
S
G1
Vehicle
20nM 50nM
CMLD011580
20nM 50nM
CR-1-31-b
e
10μM
50μM
% Unwound duplex
Vehicle
CMLD011580
CR-1-31-b
CMLD011580
CR-1-31-b

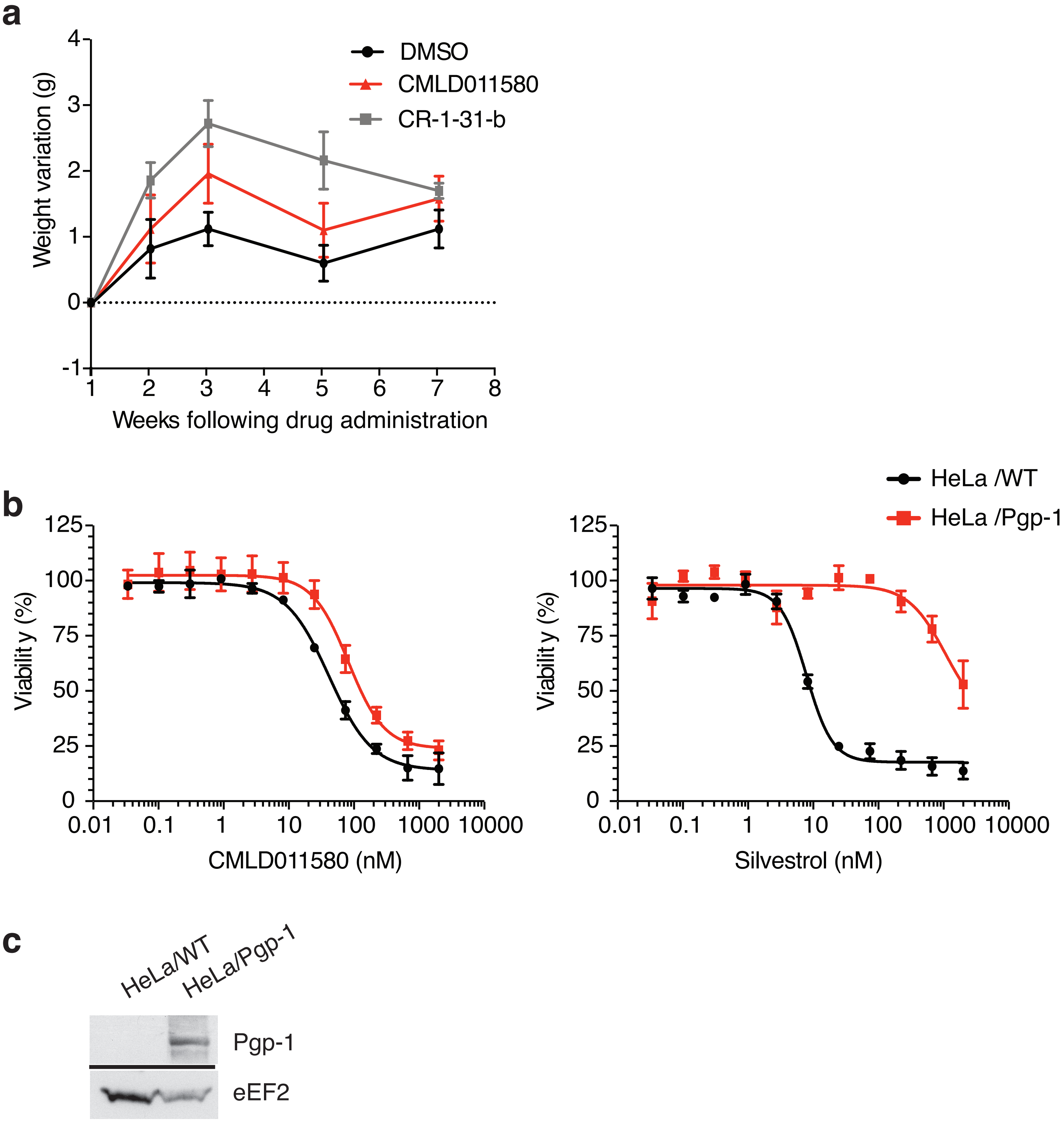
a
Weight variation (g)
DMSO
CMLD011580
CR-1-31-b
Weeks following drug administration
b
Viability (%)
CMLD011580 (nM)
Silvestrol (nM)
HeLa /WT
HeLa /Pgp-1
c
HeLa/WT
HeLa/Pgp-1
Pgp-1
eEF2

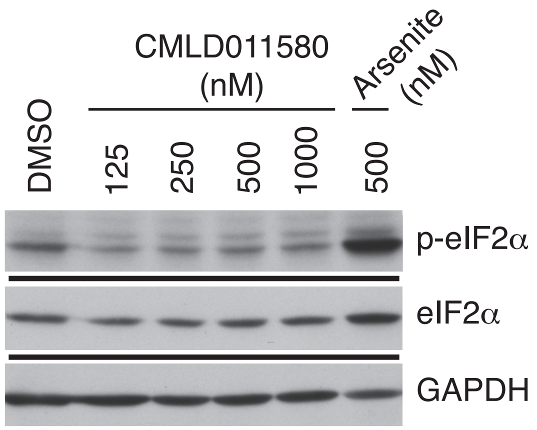
DMSO
CMLD011580
(nM)
125
250
500
1000
Arsenite
(nM)
500
p-eIF2α
eIF2α
GAPDH
